# *Strongyloides* Antigens Alter Cytokines Responses in *Toxoplasma gondii*‐Infected BeWo Trophoblast Cells

**DOI:** 10.1002/cbin.70117

**Published:** 2025-12-16

**Authors:** Fabíola Teixeira Alves, Bhrenda Carolliny Guardieiro Jesus, Maria Eduarda Silva Diniz, Luana Carvalho Luz, Priscila Silva Franco, Vanessa da Silva Ribeiro, Edson Fernando Goulart de Carvalho, Bellisa Freitas Barbosa, Henrique Tomaz Gonzaga

**Affiliations:** ^1^ Laboratório de Diagnóstico de Parasitoses, Instituto de Ciências Biomédicas Universidade Federal de Uberlândia Minas Gerais Brazil; ^2^ Laboratório de Imunofisiologia da Reprodução, Instituto de Ciências Biomédicas Universidade Federal de Uberlândia Minas Gerais Brazil

**Keywords:** cell viability, co‐infection, control strategies

## Abstract

The importance of co‐infections is not yet completely understood, and the impact and direction of their effects vary considerably. This study aimed to evaluate the role of total saline extract (TS) and excretory/secretory (ES) products of *Strongyloides venezuelensis* filarioid larvae on a maternal‐interface cell model during *Toxoplasma gondii* infection. BeWo cells were cultured and stimulated with TS antigens and ES products to evaluate their effects on cell viability, *T. gondii* proliferation, and cytokine production. Both TS and ES had no impact on BeWo cell viability and *T. gondii* proliferation. However, stimulation with the highest concentration of TS resulted in increased IL‐4 production. Additionally, IL‐6 levels significantly increased after *T. gondii* infection in all ES‐treated conditions. Increased IL‐4 and IL‐6 production was observed in comparison to the untreated control group (C) and/or between infected and uninfected cells under the same antigenic stimulation. Moreover, MIF levels increased consistently after *T. gondii* infection, regardless whether antigenic stimulation was present. Our results show that *S. venezuelensis* antigens can influence the host immune environment, altering the secretion profile of IL‐4 and IL‐6 in BeWo cells, thereby highlighting the complexity of the helminth and protozoan interaction. These studies are essential for a comprehensive understanding of responses in strongyloidiasis and their possible implications for *T. gondii* infection and disease control strategies.

## 1 Introduction

Parasitic infections represent a significant challenge to global public health, with high mortality and morbidity rates (World Health Organization 2016). The specific characteristics of each parasite influence host immune response and also alter the dynamics of each pathogen. Likewise, the immune system state of each individual is decisive for the clinical severity and outcome of parasitic diseases (Schlosser‐Brandenburg et al. 2023).

*Toxoplasma gondii* is an obligate intracellular protozoan parasite that infects a variety of cells and tissues from different hosts. Estimates indicate that around one‐third of the world population is infected by *T. gondii*, with higher incidence rates in South America, Southwest Asia, and Africa (Yan et al. 2016). In humans, toxoplasmosis is related to serious birth defects when the primary infection is acquired during the first trimester of pregnancy (Ahmed et al. 2020).

Human strongyloidiasis, caused by *Strongyloides stercoralis*, is a neglected disease and affects approximately 600 million people worldwide (Buonfrate et al. 2020). Strongyloidiasis, in general, remains asymptomatic in immunocompetent individuals; however, it may develop to fatal hyperinfection or disseminated syndromes in immunocompromised patients (Buonfrate et al. 2023).

The infection caused by *T. gondii* classically triggers a Th1‐type immune response, and requires components of innate and adaptive immunity (Khan and Moretto 2022). In contrast, species of the genus *Strongyloides* induce a Th2 type immune response, with the production of IL‐3, IL‐4, IL‐5, IL‐6, IL‐10 and IL‐13, IgM, IgG, IgA, and IgE antibodies, as well as the action of mast cells, basophils, eosinophils, and B lymphocytes (Breloer and Abraham 2017).

The simultaneous presence of multiple parasite species is commonly observed. The relevance of the interactions between these parasites for patterns of disease is still poorly understood, and analysis of co‐infection is essential, as it can affect both treatment and susceptibility to infection (Hamed et al. 2023). There is a lack of studies on the relationship between *T. gondii* and *Strongyloides*. The administration of *S. venezuelensis* antigens resulted in increased survival rates, reduced parasitic load in the lungs and small intestine, and an anti‐inflammatory profile in mice infected by *T. gondii* (Araujo et al. 2021). The aim of this study was to simulate co‐infection, evaluating the role of total saline extract (TS) and excretory/secretory (ES) products of *S. venezuelensis* filarioid larvae in a maternal‐interface cell model using human villous trophoblast cells (BeWo lineage) during *T. gondii* infection analyzing the immune response and parasite proliferation.

## 2 Materials and Methods

### 2.1 *S*. *venezuelensis* Antigenic Extract

Total saline extract (TS) from *S. venezuelensis* L3 larvae was obtained according to Gonzaga et al (Tomaz Gonzaga et al. 2011). *S. venezuelensis* L3 larvae cultures were used to obtain excretory/secretory (ES) products according to Cunha et al (Cunha et al. 2017). The protein concentration of TS or ES, after filtration (0.22 μm), was performed using the method of Lowry and collaborators (Lowry et al. 1951), modified for microplates. The concentrations of TS and ES were chosen based on preliminary experiments. A broad range of concentrations was first evaluated in cytotoxicity and proliferation assays to determine the non‐toxic, effective doses. A representative subset of these concentrations was then used for the subsequent cytokine analyses to ensure an efficient investigation.

### 2.2 Cell Viability and *T*. *gondii* Proliferation Assays

Human villous trophoblast cells (BeWo line) were obtained from the American Type Culture Collection (ATCC, Manassas, VA, USA), cultured in RPMI‐1640 medium (Cultilab, Campinas, SP, Brazil) and supplemented with 10% fetal bovine serum (FBS) (Cultilab) and antibiotics (10,000 U/mL of penicillin and 10 mg/mL of streptomycin) (Sigma Chemical Co., USA) (37°C and 5% CO_2_). BeWo cells assessed by MTT assay [3‐(4,5‐dimethylthiazol‐2‐yl)‐2,5‐diphenyltetrazolium bromide] and culture of tachyzoites (2F1 clone), which constitutively express the β‐galactosidase gene and viability of BeWo cells after stimulus with TS or ES were conducted according to the protocol described by Diniz et al (Diniz et al. 2025). The *T. gondii* proliferation in BeWo cells after stimulus with TS or ES at different concentrations (32, 8, 2, 0.5, and 0.125 μg/mL) was verified using the β‐galactosidase assay, as described by Diniz et al. (Diniz et al. 2025). The number of tachyzoites was calculated based on a standard curve containing free tachyzoites from 1 × 10^6^ to 1.5625 × 10^3^ parasites. Three independent experiments with eight replicates were performed.

Cells were divided into groups as follows: Negative control (C)—infected BeWo cells maintained only in medium; positive control (SP)—cells treated with sulfadiazine + pyrimethamine; and antigen‐treated groups: cells (infected or not) stimulated with TS or ES at different concentrations.

### 2.3 Cytokine Measurement by Indirect ELISA

The human cytokines IL‐4, IL‐6, IL‐8, IL‐10, and MIF were measured in supernatants collected from BeWo cell cultures treated with 32, 8, or 2 μg/mL of TS or ES, by the Enzyme‐linked immunosorbent assay—ELISA (according to manufacturer's instructions—BD Biosciences; R&D Systems). Cytokine concentrations were expressed in pg/mL. The detection limits for each cytokine were: IL‐4: 7.81 pg/mL; IL‐6: 4.7 pg/mL; IL‐8: 3.125 pg/mL; IL‐10: 7.81 pg/mL; and MIF: 31.25 pg/mL. Three independent experiments with four replicates were performed.

### 2.4 Statistical Analysis

The data obtained were initially analyzed to identify outliers using the ROUT method, with *Q* = 10%. All datasets were tested for normality (Shapiro–Wilk test) and homogeneity of variances (Brown–Forsythe test). Data were analyzed using a one‐way ANOVA followed by Dunnett multiple comparisons post‐test; or Welch ANOVA with a Dunnett T3 post‐test as an alternative. Multiple comparisons test was performed to compare with the negative control group (C): (a) cell viability in cells treated with 64 to 0.125 μg/mL of TS or ES; (b) *T. gondii* proliferation (SP, 32, 8, 2, 0.5 and 0.125 μg/mL); and (c) cytokine level of each condition (SP, 32, 8 and 2 μg/mL). The mean of control group C was used as a correction factor for *T. gondii* proliferation in TS and ES‐treated cells, for subsequent 2 to 2 concentration comparison. Also, for cytokines, the infected and uninfected conditions were compared using the Student *t*‐test. All data were expressed as mean ± standard deviation (SD). Analyzes were performed using GraphPad Prism Version 10 software, and *p* values < 0.05 were considered statistically significant.

## 3 Results

### 3.1 Total Saline and Excretory/Secretory (ES) Products of *S*. *venezuelensis* Did Not Alter the Viability of BeWo Cells

The cell viability assay (MTT) was performed to investigate the potential toxicity of TS or ES antigens of *S. venezuelensis* L3 larvae in BeWo cells, in a wide range of concentrations (64; 32; 16; 8; 4; 2 and 0.5 μg/mL). The results indicated that both antigenic preparations, regardless of concentration, showed no significant difference in the viability of BeWo cells when compared to the control group, composed of untreated cells (data not shown).

### 3.2 Total Saline and Excretory/Secretory (ES) Products of *S*. *venezuelensis* at Different Concentrations Did Not Affect the *T*. *gondii* Intracellular Proliferation in BeWo Cells

**Figure 1:**
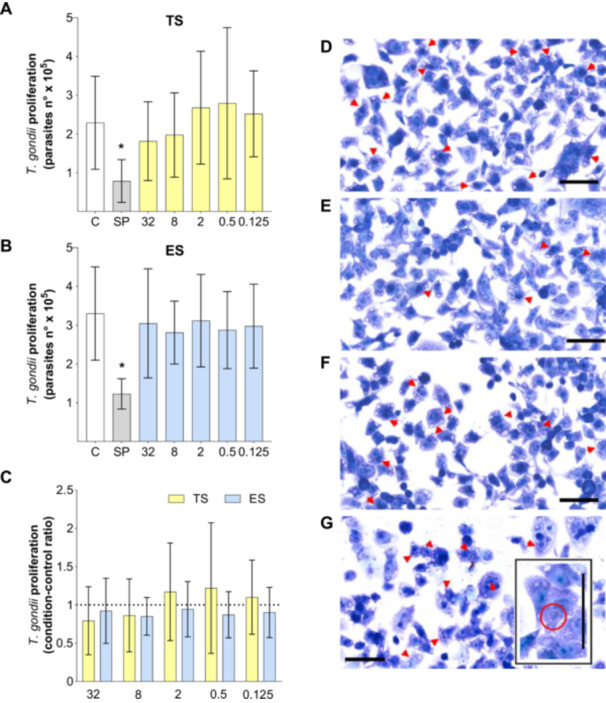
(A–C) *Toxoplasma gondii* proliferation: quantification of intracellular tachyzoites by the enzymatic reaction of β‐galactosidase activity. BeWo cells infected by *T. gondii* were treated for 24 h with total saline extract (TS, A) or excretory/secretory (ES) products (ES, B) of *S. venezuelensis* L3, at concentrations of 32, 8, 2, 0.5, and 0.125 μg/mL. Experimental controls: negative (C)—infected BeWo cells maintained only in medium, and positive (SP) addition of sulfadiazine + pyrimethamine (SP). Data were expressed as mean ± standard deviation (A and B). In C, the mean of the negative control (C) was used as a correction factor for *T. gondii* proliferation in the different conditions of TS or ES. Significant differences were determined by: One‐way ANOVA, followed by Dunnet post‐test (A and B); or Student *t‐*test (C). (*) indicates *p* < 0.05, signifying a difference in relation to the negative control (C) for panels A and B. For panel C, no statistically significant differences were observed between antigenic preparations. (D–G) Photomicrographs illustrate the proliferation assay results: C (D), SP (E), TS (F, 32 μg/mL), and ES (G, 32 μg/mL). Arrowheads point to parasitophorous vacuole. Toluidin blue; bar scale—60 μm. Three independent experiments with eight replicates were performed.

BeWo cells were infected and, after 3 h, treated with TS or ES antigens at the following concentrations 32, 8, 2, 0.5, and 0.125 μg/mL for 24 h. The treatment with different concentrations of TS or ES did not affect *T. gondii* proliferation (Figure 1A,B). Additionally, no difference in *T. gondii* proliferation or condition/control ratio, which represents the relative parasite proliferation in antigen‐treated cells compared to the infected control (C), were observed between antigenic preparations, at the same concentrations (Figure 1A–C). Figure 1D–G shows representative images of the assay under control conditions (C and SP) and at a concentration of 32 μg/mL of TS or ES.

### 3.3 Stimulation With Strongyloides Antigens Alter the Secretion Profile of IL‐4 and IL‐6 in *T*. *gondii* Infected BeWo Cells

**Figure 2:**
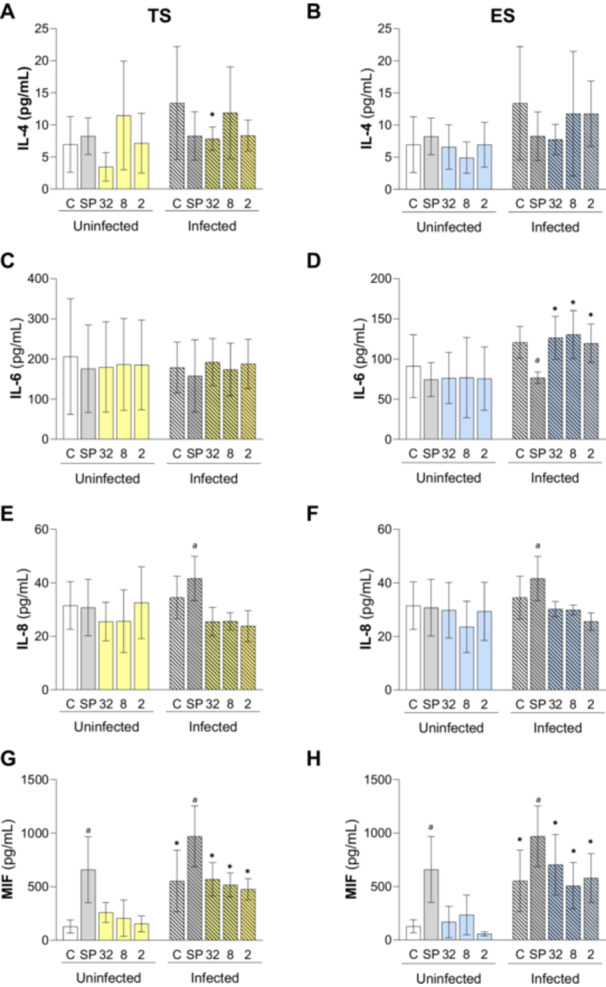
Analysis of the cytokine profile IL‐4 (A, B), IL‐6 (C, D), IL8 (E, F) and MIF (G, H) secreted by BeWo cells. Supernatants from BeWo cells uninfected or infected: negative control (C); positive control (SP— sulfadiazine + pyrimethamine); treated (TS or ES—32, 8, or 2 μg/mL), were subjected to cytokine measurement by ELISA assay. The results were expressed in pg/mL. Differences between groups were analyzed by One‐Way ANOVA test followed by Dunnet post‐test; letter ‘a’ indicates difference (*p* < 0.05) in relation to control. The infected and uninfected conditions were compared using the Student *t‐*test (*p < 0.05). Three independent experiments with four replicates were performed. ANOVA, Analysis of variance; ATCC, American Type Culture Collection; C, Negative control; ELISA, Enzyme‐linked immunosorbent assay; ES, Excretory/secretory products of *S. venezuelensis* L3 larvae; FBS, Fetal bovine serum; IFN‐γ, Interferon gamma; IL, Interleukin; MIF, Macrophage migration inhibitory factor; MTT, 3‐(4,5‐dimethylthiazol‐2‐yl)‐2,5‐diphenyltetrazolium bromide; RPMI‐1640, Roswell Park Memorial Institute‐1640 medium; SD, Standard deviation; SP, Sulfadiazine + pyrimethamine; Th1, T helper type 1; Th2, T helper type 2; TS, Total saline extract of *S. venezuelensis* L3 larvae.

The supernatants from the different culture conditions of BeWo cells with TS or ES antigens of *S. venezuelensis* L3 larvae and *T. gondii* were subjected to the ELISA assay to verify the profile of secreted cytokines (Figure 2). Regarding IL‐4 production, for ES, no significant difference was observed between the infected and non‐infected groups and the respective negative control (Figure 2B). However, it was seen at the higher concentration of TS (32 μg/mL) a greater production of IL‐4 in infected cells (Figure 2A). IL‐6 production increased significantly after *T. gondii* infection at all ES concentrations (Figure 2C). For cells treated with TS and ES, there was no difference in the production of IL‐6 between the untreated control and C and SP before and after infection with *T. gondii* (Figure 2C,D). Regarding the production of IL‐8, no significant difference was observed in any of the concentrations for both antigens; however an increase in IL‐8 in SP control after infection (Figure 2E,F). MIF detection showed a significant increase after *T. gondii* infection in the untreated control and at all concentrations of ES or TS (Figure 2G,H). Regarding IL‐10, concentrations of this cytokine were consistently below the detection limit across all experimental conditions.

## 4 Discussion

It is widely known that co‐infection situations are naturally present in endemic regions; however, these cases are often unreported. The modulation of the immune response in parasitic co‐infections is a growing field of study, as infection by a parasite can influence the immune response and the pathogenic outcome of subsequent infections, generating synergistic or antagonistic effects on the host (Xu et al. 2020).

During pregnancy, the maternal immune system undergoes profound adaptations to promote fetal tolerance, which may also increase susceptibility to infections. Pregnancy immunology is now recognized as a dynamic process: pro‐inflammatory responses predominate during the first and third trimesters, whereas an anti‐inflammatory state characterizes the second trimester (Aghaeepour et al. 2017; Abu‐Raya et al. 2020). Within this context, a Th1‐to‐Th2 shift favors humoral over cytotoxic responses, a modulation essential for fetal survival but detrimental to the control of intracellular pathogens such as *T. gondii*. Conversely, this same Th2‐biased environment creates permissive conditions for helminths like *Strongyloides*, facilitating their persistence during gestation (Wikman‐Jorgensen et al. 2021).

In our study, BeWo cells exposed to *S. venezuelensis* antigens did not show significant changes in their viability. In addition, we treated BeWo cells infected with *T. gondii* for 24 h with different concentrations of total saline and excretory/secretory (ES) products of *S. venezuelensis*, which also did not affect *T. gondii* replication.

In contrast to our results, an in vivo non‐congenital infection model showed a significant reduction in parasite load in the lungs and small intestine when the saline extract of *S. venezuelensis* was administered after *T. gondii* infection (Araujo et al. 2021). Interestingly, *Trypanosoma cruz*i infection reduced *T. gondii* growth in BeWo cells, but triggered an increase in tachyzoites replication in human placental explants, highlighting that co‐infection with these parasites can induce different phenomena in a dependent manner of cell or tissue types (de Souza et al. 2023). Also, a recent study demonstrated that the prevalence of co‐infections with *T. cruzi* and helminths was 8.1% in Argentina, with approximately 2% attributed to *S. stercoralis*, suggesting a possible facilitation of *Trypanosoma* transmission triggered by helminthic infections (Enriquez et al. 2024). In line with this, rats co‐infected with *T. brucei* and *S. ratti* presented reduced blood parasitemia and ameliorated pathogenic effects, showing that *Strongyloides* had ability to control *Trypanosoma* infection (Onah et al. 2004). All these findings indicate that helminths, such as *Strongyloides* (and other protozoa), or their antigens can differentially modulate the *infection* by protozoans, such as *T. gondii*. Fully understanding the mechanisms underlying these interactions in a variety of settings—particularly in the context of gestational infection and congenital transmission—is essential for a comprehension of responses to strongyloidiasis and their potential implications for *T. gondii* infection.

Helminthic products applied against *T. gondii* proliferation demonstrated promising effects. For example, Khan and colleagues (Khan et al. 2008) reported that early infection with the mouse intestinal nematode *Heligsomosoides polygyrus* can suppress the response of T CD4^+^ and CD8^+^ cells, as well as the production of IL‐12 and IFN‐γ during *T. gondii* infection. In a murine model of *T. gondii* and *Fasciola hepatica* co‐infection, the production of *T. gondii*‐specific Th1 cytokines was not suppressed by *F. hepatica* infection. However, *T. gondii* infection inhibited the ability of splenocytes from *F. hepatica‐*infected mice to produce Th2 cytokines (Miller et al. 2009).

*Toxoplasma gondii* infection is classically characterized by a strong Th1 response, while *Strongyloides* triggers the production of Th2‐type cytokines. As these parasites induce distinct immune response profiles, we examined whether treatment with TS or ES might interfere with the levels of cytokines produced by BeWo cells. *T. gondii* infection increased the levels of IL‐4 (32 μg/mL, TS) and IL‐6 (32, 8, and 2 μg/mL ES) after TS or ES stimulus, respectively. Even with the similarity to the levels of the untreated control group (which only increased MIF after infection), stimulation with *Strongyloides* antigens appears to favor the secretion profile of IL‐4, IL‐6, and MIF in some conditions.

In contrast to our findings, Araujo and collaborators (Araujo et al. 2021) reported higher levels of IL‐6 in serum and small intestine of mice infected by *T. gondii*, but these levels were reduced post‐treatment with *S. venezuelensis* L3 antigens. For IL‐4, no differences were observed, even after treatment with *S. venezuelensis*. Our previous studies showed that IL‐6 and MIF are important cytokines to control *T. gondii* proliferation in BeWo cells (Barbosa et al. 2014, 2015; da Silva et al. 2017; Oliveira et al. 2021). In this context, it is possible that these high levels of IL‐6 and MIF after TS or ES stimulus could contribute to avoiding the exacerbated growth of tachyzoites, justifying the absence of changes in parasite load in BeWo cells. At the same time, the elevated levels of IL‐4 can be associated with the prevention of an exacerbated proinflammatory scenario, maintaining the regulatory profile favorable to embryo development. These discrepancies underscore the importance of the cellular context and experimental model (in vitro vs. in vivo) in influencing immune outcomes. Our data also suggests that *S. venezuelensis* antigens may exert distinct immunomodulatory effects at the maternal‐fetal interface, which is particularly relevant during pregnancy, when immune regulation is critical. Therefore, our findings enhance the understanding of how helminth‐derived antigens can affect cytokine responses during *T. gondii* infection in cell types relevant to congenital transmission.

Overall, the modulation of cytokines in BeWo cells in response to *S. venezuelensis* antigens suggests a complex interplay that may influence infection outcomes during pregnancy—either by reducing inflammation or by compromising parasite control. Future studies are necessary to verify the mechanisms of action, possibly triggered by Strongyloides, that are able to modulate the immune response at the human maternal‐fetal interface.

## 5 Conclusion

Our results suggest that *S. venezuelensis* antigens, though not directly affecting parasite proliferation or host cell viability, can influence the host immune environment altering the secretion profile of IL‐4 and IL‐6 in a parasite‐specific and antigen‐dependent manner. This immunomodulatory potential may have implications for understanding host–pathogen interactions in the context of co‐infections during pregnancy.

This study presents some limitations, such as the use of an in vitro model and the potential variability in the antigenic preparations used. Further studies with different models are needed to explain the processes underlying these relationships for a complete understanding of responses in strongyloidiasis and their possible implications for *T. gondii* infection and disease control strategies.

## Conflicts of Interest

The authors declare no conflicts of interest.

## Data Availability

The data that support the findings of this study are available from the corresponding author upon reasonable request.

## References

[cbin70117-bib-0001] Abu‐Raya, B. , C. Michalski , M. Sadarangani , and P. M. Lavoie . 2020. “Maternal Immunological Adaptation During Normal Pregnancy.” Frontiers in Immunology 11: 575197. 10.3389/fimmu.2020.575197. 33133091 PMC7579415

[cbin70117-bib-0002] Aghaeepour, N. , E. A. Ganio , D. Mcilwain , et al. 2017. “An Immune Clock of Human Pregnancy.” Science Immunology 2, no. 15: eaan2946. 10.1126/sciimmunol.aan2946. 28864494 PMC5701281

[cbin70117-bib-0003] Ahmed, M. , A. Sood , and J. Gupta . 2020. “Toxoplasmosis in Pregnancy.” European Journal of Obstetrics & Gynecology and Reproductive Biology 255: 44–50. 10.1016/j.ejogrb.2020.10.003. 33075679

[cbin70117-bib-0004] Araujo, E. C. B. , Y. Cariaco , M. P. O. Almeida , et al. 2021. “Beneficial Effects of *Strongyloides venezuelensis* Antigen Extract in Acute Experimental Toxoplasmosis.” Parasite Immunology 43, no. 4: e12811. 10.1111/pim.12811. 33247953

[cbin70117-bib-0005] Barbosa, B. F. , J. B. Lopes‐Maria , A. O. Gomes , et al. 2015. “IL10, TGF Beta1, and IFN Gamma Modulate Intracellular Signaling Pathways and Cytokine Production to Control *Toxoplasma gondii* Infection in BeWo Trophoblast Cells.” Biology of Reproduction 92, no. 3: 82. 10.1095/biolreprod.114.124115. 25673564

[cbin70117-bib-0006] Barbosa, B. F. , L. Paulesu , F. Ietta , et al. 2014. “Susceptibility to *Toxoplasma gondii* Proliferation in BeWo Human Trophoblast Cells Is Dose‐Dependent of Macrophage Migration Inhibitory Factor (MIF), via ERK1/2 Phosphorylation and Prostaglandin E2 Production.” Placenta 35, no. 3: 152–162. 10.1016/j.placenta.2013.12.013. 24433846

[cbin70117-bib-0007] Breloer, M. , and D. Abraham . 2017. “ *Strongyloides* Infection in Rodents: Immune Response and Immune Regulation.” Parasitology 144, no. 3: 295–315. 10.1017/S0031182016000111. 26905057

[cbin70117-bib-0008] Buonfrate, D. , D. Bisanzio , G. Giorli , et al. 2020. “The Global Prevalence of *Strongyloides stercoralis* Infection.” Pathogens 9: 468. 10.3390/pathogens9060468. 32545787 PMC7349647

[cbin70117-bib-0009] Buonfrate, D. , R. S. Bradbury , M. R. Watts , and Z. Bisoffi . 2023. “Human Strongyloidiasis: Complexities and Pathways Forward.” Clinical Microbiology Reviews 36: e0003323. 10.1128/cmr.00033-23. 37937980 PMC10732074

[cbin70117-bib-0010] Cunha, R. A. , E. F. G. de Carvalho , J. E. N. de Sousa , and J. M. Costa‐Cruz . 2017. “Excretory/Secretory Antigens of *Strongyloides venezuelensis* Applied to IgG Detection in Human Strongyloidosis.” Parasitology International 66, no. 5: 671–676. 10.1016/j.parint.2017.07.001. 28705595

[cbin70117-bib-0011] Diniz, M. E. S. , F. T. Alves , B. C. G. Jesus , et al. 2025 Mar. “ *Taenia crassiceps* cysticerci Antigenic Extract Controls *Toxoplasma gondii* Proliferation in Human Trophoblast Cells and Upregulates IL‐10 Production.” Microbial Pathogenesis 200: 107353. 10.1016/j.micpath.2025.107353. 39892833

[cbin70117-bib-0012] Enriquez, G. F. , N. P. Macchiaverna , G. Garbossa , et al. 2024. “Humans Seropositive for *Trypanosoma cruzi* Co‐Infected With Intestinal Helminths Have Higher Infectiousness, Parasitaemia and Th2‐Type Response in the Argentine Chaco.” Parasites & Vectors 17, no. 1: 340. 10.1186/s13071-024-06401-7. 39135121 PMC11320973

[cbin70117-bib-0013] Hamed, E. F. A. , N. E. Mostafa , E. M. Fawzy , et al. 2023. “ *Toxoplasma gondii* suppresses Th2‐induced by *Trichinella* Spiralis Infection and Downregulates Serine Protease Genes Expression: A Critical Role in Vaccine Development.” Iranian Journal of Parasitology 18, no. 2: 172–181. 10.18502/ijpa.v18i2.13183. 37583627 PMC10423907

[cbin70117-bib-0014] Khan, I. A. , R. Hakak , K. Eberle , P. Sayles , L. M. Weiss , and J. F. Urban . 2008. “Coinfection With *Heligmosomoides polygyrus* Fails to Establish CD8+ T‐Cell Immunity Against *Toxoplasma gondii* .” Infection and Immunity 76, no. 3: 1305–1313. 10.1128/IAI.01236-07. 18195022 PMC2258819

[cbin70117-bib-0015] Khan, I. A. , and M. Moretto . 2022. “Immune Responses to *Toxoplasma gondii* .” Current Opinion in Immunology 77: 102226. 10.1016/j.coi.2022.102226. 35785567

[cbin70117-bib-0016] Lowry, O. , N. Rosebrough , A. L. Farr , and R. Randall . 1951. “Protein Measurement With the Folin Phenol Reagent.” Journal of Biological Chemistry 193: 265–275. 14907713

[cbin70117-bib-0017] Miller, C. M. D. , N. C. Smith , R. J. Ikin , N. R. Boulter , J. P. Dalton , and S. Donnelly . 2009. “Immunological Interactions Between 2 Common Pathogens, Th1‐Inducing Protozoan *Toxoplasma gondii* and the Th2‐Inducing Helminth *Fasciola hepatica* .” PLoS One 4, no. 5: e5692. 10.1371/journal.pone.0005692. 19478853 PMC2682559

[cbin70117-bib-0018] Oliveira, F. C. , R. J. Silva , M. Ribeiro , et al. 2021. “ERK1/2 Phosphorylation and IL‐6 Production Are Involved in the Differential Susceptibility to *Toxoplasma gondii* Infection in Three Types of Human (Cyto/Syncytio/Extravillous) Trophoblast Cells.” Tissue & Cell 72: 101544. 10.1016/j.tice.2021.101544. 33892398

[cbin70117-bib-0019] Onah, D. N. , I. W. Onyenwe , J. I. Ihedioha , and O. S. Onwumere . 2004. “Enhanced Survival of Rats Concurrently Infected With *Trypanosoma brucei* and *Strongyloides ratti* .” Veterinary Parasitology 119, no. 2–3: 165–176. 10.1016/j.vetpar.2003.08.007. 14746976

[cbin70117-bib-0020] Schlosser‐Brandenburg, J. , A. Midha , R. M. Mugo , et al. 2023. “Infection With Soil‐Transmitted Helminths and Their Impact on Coinfections.” Frontiers in Parasitology 2: 1197956. 10.3389/fpara.2023.1197956. 39816832 PMC11731630

[cbin70117-bib-0021] da Silva, R. J. , A. O. Gomes , P. S. Franco , et al. 2017. “Enrofloxacin and Toltrazuril Are Able to Reduce *Toxoplasma gondii* Growth in Human BeWo Trophoblastic Cells and Villous Explants From Human Third Trimester Pregnancy.” Frontiers in Cellular and Infection Microbiology 7: 340. 10.3389/fcimb.2017.00340. 28798905 PMC5526852

[cbin70117-bib-0022] de Souza, G. , S. C. Teixeira , A. F. Fajardo Martínez , et al. 2023. “ *Trypanosoma cruzi* P21 Recombinant Protein Modulates *Toxoplasma gondii* Infection in Different Experimental Models of the Human Maternal‐Fetal Interface.” Frontiers in Immunology 14: 1243480. 10.3389/fimmu.2023.1243480. 37915581 PMC10617204

[cbin70117-bib-0023] Tomaz Gonzaga, H. , V. da Silva Ribeiro , J. Pereira cunha‐Júnior , M. Tiduko Ueta , and J. M. Costa‐Cruz . 2011. “Usefulness of Concanavalin‐A Non‐Binding Fraction of *Strongyloides venezuelensis* Larvae to Detect IgG and IgA in Human Strongyloidiasis.” Diagnostic Microbiology and Infectious Disease 70, no. 1: 78–84. 10.1016/j.diagmicrobio.2011.01.016. 21513846

[cbin70117-bib-0024] Wikman‐Jorgensen, P. , A. Requena‐Méndez , and J. Llenas‐García . 2021. “A Review on Strongyloidiasis in Pregnant Women.” Research and Reports in Tropical Medicine 12: 219–225. 10.2147/RRTM.S282268. 34584485 PMC8464358

[cbin70117-bib-0025] World Health Organization . “Global Health Estimates 2016: Deaths by Cause, Age, Sex, by Country and by Region, 2000–2016.” 2016.

[cbin70117-bib-0026] Xu, F. , R. Cheng , S. Miao , et al. 2020. “Prior *Toxoplasma gondii* Infection Ameliorates Liver Fibrosis Induced by *Schistosoma japonicum* Through Inhibiting th2 Response and Improving Balance of Intestinal Flora in Mice.” International Journal of Molecular Sciences 21, no. 8: 2711. 10.3390/ijms21082711. 32295161 PMC7216211

[cbin70117-bib-0027] Yan, C. , L. J. Liang , K. Y. Zheng , and X. Q. Zhu . 2016. “Impact of Environmental Factors on the Emergence, Transmission and Distribution of *Toxoplasma gondii* .” Parasites & Vectors 9: 137. 10.1186/s13071-016-1432-6. 26965989 PMC4785633

